# Filtration of the Microalga *Amphidinium carterae* by the Polychaetes *Sabella spallanzanii* and *Branchiomma luctuosum*: A New Tool for the Control of Harmful Algal Blooms?

**DOI:** 10.3390/microorganisms10010156

**Published:** 2022-01-12

**Authors:** Loredana Stabili, Margherita Licciano, Adriana Giangrande, Carmela Caroppo

**Affiliations:** 1 Department of Biological and Environmental Sciences and Technology, University of Salento, 73100 Lecce, Italy; margherita.licciano@unisalento.it (M.L.); adriana.giangrande@unisalento.it (A.G.); 2 Water Research Institute (IRSA), Taranto Section CNR, 74121 Taranto, Italy

**Keywords:** harmful algal blooms, bioremediation, filtration activity, polychaetes, dinoflagellates

## Abstract

Harmful algal blooms (HABs) are extreme biological events representing a major issue in marine, brackish, and freshwater systems worldwide. Their proliferation is certainly a problem from both ecological and socioeconomic contexts, as harmful algae can affect human health and activities, the marine ecosystem functioning, and the economy of coastal areas. Once HABs establish, valuable and environmentally friendly control actions are needed to reduce their negative impacts. In this study, the influence exerted by the filter-feeding activity of the two sabellid polychaetes *Branchiomma luctuosum* (Grube) and *Sabella spallanzanii* (Gmelin) on a harmful dinoflagellate was investigated. Clearance rates (C) and retention efficiencies were estimated by employing the microalga *Amphidinium carterae* Hulburt. The C_max_ was 1.15 ± 0.204 L h^−1^ g^−1^ DW for *B. luctuosum* and 0.936 ± 0.151 L h^−1^ g^−1^ DW for *S. spallanzanii*. The retention efficiency was 72% for *B. luctuosum* and 68% for *S. spallanzanii*. Maximum retention was recorded after 30 min for both species. The obtained results contribute to the knowledge of the two polychaetes’ filtration activity and to characterize the filtration process on harmful microalgae in light of the protection of water resources and human health. Both species, indeed, were extremely efficient in removing *A. carterae* from seawater, thus suggesting their employment as a new tool in mitigation technologies for the control of harmful algae in marine environments, as well as in the aquaculture facilities where HABs are one of the most critical threats.

## 1. Introduction

Marine phytoplankton forms the base of the food webs, but some species are considered “harmful” as they produce toxins that negatively affect human health, or blooms with deleterious effects on aquatic ecosystems, fisheries, aquaculture, and tourism [1,2]. The term “Harmful Algal Blooms” (HABs) is used to describe all of these events (e.g., [3]). HABs are natural phenomena that, in recent years, have increased in occurrence and spread worldwide [4], and also in relation to an enhanced scientific knowledge and an intensification of monitoring programs, especially in aquaculture sites [5].

Determining the causative factors for HABs is complex. Many studies have been carried out to understand this ecological phenomenon, but the mechanisms responsible for the bloom formation are still not fully understood and their prevention is a highly difficult task [6]. In fact, not only eutrophication, but also other causes, can be responsible for the expansion and intensification of HABs, such as human-mediated introduction of alien harmful species, climatic variability, expanded utilization of coastal waters for aquaculture, and habitat modification [7,8]. Preventive measures, such as nutrient input control, are necessary to preclude the occurrence of high biomass HABs. However, HABs may continue to increase due to warming and rising CO_2_, according to the 2019 IPCC [9]. While the exact scenarios are unknown and more studies should be conducted, the fact that HABs are, in part, natural events requires us to address methods to mitigate their impacts. Effective and environmentally friendly control actions have become a priority to suppress them [4,5]. Biological methods represent promising options to control HABs, as they usually do not cause secondary pollution and can be species-specific [10]. They usually use enzymolysis or parasitism effects on HAB species and nutrient competition, or allelopathic effects of macroalgae or seagrass, or grazing by marine protozoa, zooplankton, and filter-feeding shellfish [4]. Marine invertebrate filter feeders have been shown to extract large quantities of phytoplankton from the water, thus having a pronounced grazing impact on the phytoplankton communities and biomass in many shallow marine areas. The diversity of organisms involved in the filtration process thus ensures water filtration, the use of plankton, and water self-purification [11,12,13]. In coastal marine ecosystems, these filter feeders constitute a large component both in terms of biomass and abundance and, due to their intense filtering capacity, these organisms could potentially process large volumes of water daily. Several researchers have studied the filtration activities of different zoological groups, such as ascidians, polychaetes, bivalves, and sponges [14,15,16,17,18,19,20,21,22,23,24], with different types and concentrations of particles [11,25]. 

Polychaetes species belonging to the Sabellidae family are characterized by the presence of a branchial crown, which protrudes out of the tube they inhabit to collect and sort material of different sizes. Small particles are ingested, while large ones are rejected into the water [26,27]. Several studies have been focused on the filtering organs and particle capture mechanisms in sabellids [14,15,16,20,21,22,23,24,28,29,30,31,32,33,34,35]. Most of the field and laboratory studies on filter feeder polychaetes have been carried out employing phytoplankton as a trophic source [29,33,34]. In particular, Clapin [29] has shown that the sabellid *Sabella spallanzanii* (Gmelin), when settled on artificial structures at about 5 m depth, is able to influence the phytoplankton level through the filtration process, filtering 23.4 × 10^3^ L m^−2^ day^−1^ (biomass = 258 g DW m^−2^), and, finally, to ensure, for almost five times a day, a turnover of the overlying water column.

In the present paper, we investigate the filtration process of two sabellid species, *S. spallanzanii* and *Branchiomma luctuosum*, on the potential toxic dinoflagellate species *Amphidinium carterae*. We characterized the filtration process of these two sabellid species and evaluated their clearance rate and retention efficiency. The filtration capability of these two species was previously studied by using bacterioplankton as a trophic source [14,15,16].

*Amphidinium carterae* is a high-biomass producer [36,37,38], as well as a producer of more than 20 secondary metabolites that have demonstrated haemolytic, cytotoxic, ichthyotoxic, and antifungal activities [39,40].

Until now, mitigation measures tested for the blooms of this microalga were chemical controls using modified sands, clay, and soil [41,42,43]. The obtained results are discussed in light of the potential employment of the investigated polychaete species as bioremediators of harmful microalgae, effective in protecting water resources and human health in coastal waters, as well as in the field of the aquaculture.

## 2. Material and Methods

### 2.1. Polychaete Sampling

**Figure 1:**
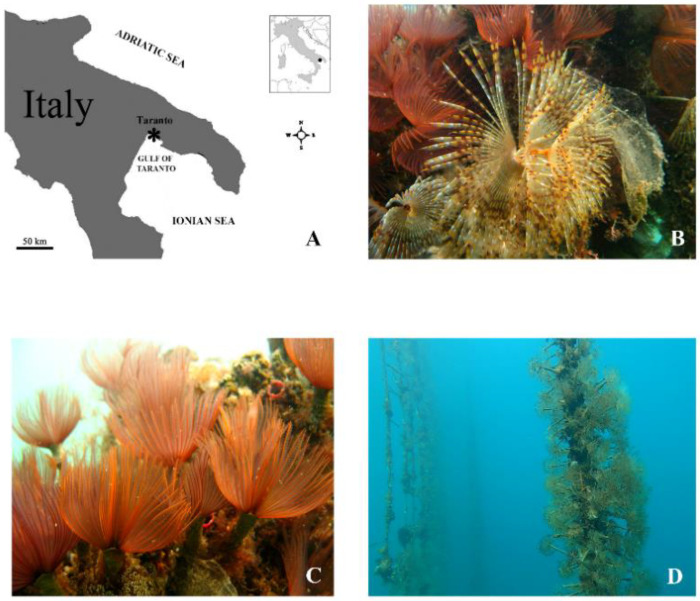
(**A**) Map of the sampling site in the Gulf of Taranto (Mediterranean Sea, Ionian Sea, Italy). (**B**) Specimen of the polychaete *Sabella spallanzanii*. (**C**) Specimens of the polychaete *Branchiomma luctuosum*. (**D**) Rearing trials of the polychaetes in the integrated multitrophic aquaculture (IMTA) system realized in the Gulf of Taranto. Polychaetes were arranged in polypropylene nets, which were hung vertically within a typical long-line system.

Adult specimens of *S. spallanzanii* and *B. luctuosum* were sampled in the Gulf of Taranto (Mediterranean Sea, Ionian Sea, Italy), where rearing trials were realized in an integrated multitrophic aquaculture (IMTA) system equipped with fish cages within the framework of the Remedia Life Project (LIFE16 ENV/IT/000343). The experimental design of the project consisted of an IMTA system including six fish cages, where the waste restoration was realized by the presence of some marine organisms, including the selected filter feeder polychaetes, acting as bioremediators. Polychaetes were obtained from natural recruitment on plastic nets, used as collectors, immersed in the fish farm around the fish cages suspended at about 12 m depth (Figure 1). After their collection, the worms were transferred to the laboratory and prepared for the filtration experiments. Prior to experiments, as already reported by Licciano et al. [14], epibionts were removed from the worm tubes, then 12 individuals of similar tube length were selected for each species, placed in an aerated aquarium equipped with 25 L of sterile-filtered sea water (SFSW) (0.22 µm pore size filters, Millipore), which was daily replaced, and kept in a temperature-controlled room (22 °C) for 48 h. After starvation, individuals of *S. spallanzanii* and *B. luctuosum* were separately utilized for the filtration experiments.

### 2.2. Microalgal Culture Conditions

The experiments were carried out using a strain of the dinoflagellate, *A. carterae,* previously isolated from the Gulf of Taranto [44,45]. Microalgal cultures were grown in f/2 medium [46]. Culture media was prepared with Gulf of Taranto seawater (salinity of 38‰) filtered through glass fiber filters (Whatman GF/C) and then autoclaved. All microorganisms were grown in 1000 mL Erlenmeyer flasks with 500 mL of f/2 medium and kept in optimal growth conditions in a thermostatic chamber at 20 ± 1 °C under an illumination cycle of 12D:12N (100 micromol photons m^−2^ s^−1^) and stirred at 100 rpm by means of a mechanical stirrer.

For the filtration experiments, microalgae were collected after thirty days of growth (exponential phase). Before biological tests, microalgal cultures were counted using a 1-mL Sedgwick Rafter chamber, and then appropriate aliquots were added to the experimental beakers to obtain the required final concentrations of *A. carterae* corresponding to 6 × 10^3^ cells mL^−1^.

### 2.3. Amphidinium carterae Retention and Clearance Rate Calculation

In order to evaluate the clearance rate (C) and the retention efficiency (R) of *A. carterae* by *S. spallanzanii* and *B. luctuosum*, two separate experiments were carried out.

For each species, six starved worms were individually placed in six beakers filled with 10 L of SFSW (treatments), and six beakers were filled only with SFSW and employed as controls, for a total of 12 beakers. Just prior to the beginning of the experiments, an *A. carterae* suspension of a known concentration (2.2 × 10^5^ cells mL^−^^1^) was added to both the treatment and control beakers so that the final concentration was about 6 × 10^3^ cells mL^−1^ for both sabellid species. The retention of *A. carterae* cells by the two investigated filter feeders was assessed by evaluating the removal of algal cells from SFSW over 4 h at a temperature of 22 °C. Aliquots of SFSW (10 mL) were aseptically collected from the treatment and control beakers every 15 min within the first hour and every 30 min during the following 3 h, for a total of 11 sampling times. In order to determine the phytoplankton abundances, from each beaker at the end of each sampling time, the aliquots of SFSW were immediately fixed with an acid Lugol’s iodine solution to a final concentration of 1% and stored at 4 °C until analysis. *Amphidinium* counts were performed by using an inverted microscope (Labovert FS Leitz) equipped with phase contrast and following the Utermöhl method [47]. Three replicates were prepared from each aliquot. At each time, a mean value from the three replicates was computed. For each species, the retention efficiency R was calculated as a percentage for the difference in algal abundances at each sampling time by the following equation:

*R (%) = 100 × [(c_0_ − c_t_)/c_0_]* (1)

where *c_0_* is the initial algal concentration in the experimental beakers and *c_t_* is the algal concentration at the end of time *t*.

In order to determine the dry weights (g) of the employed filter feeders, at the end of the experiments, each worm was drawn out of the tube, dried at 60 °C for 24 h, and then weighed.

Clearance rates were assessed according to Coughlan [48] by evaluating the algal removal in the treatment beakers as a function of time. Taking into account the predictable shifts between feeding activity and rest during the filtration process of the worms, recognizable by the intrusion of the branchial crown within the tube, for each species, C was calculated within each sampling time as the mean ± standard deviation (SD) of the C values recorded in all the treatment beakers at each time point and expressed in liters per hour, per gram, of dry worm tissue (L h^−1^ g^−1^ DW). Furthermore, according to Navarro and Widdows [17], the maximum clearance rate (C_max_) was also calculated for each species as the mean ± SD of the highest C values recorded in each treatment beaker during the entire experimental period (4 h).

### 2.4. Statistical Analysis

Analysis of variance was used to assess significant variations in *A. carterae* abundance in the treatment and control beakers. The experimental design consisted of two factors: polychaetes (Po, two levels, i.e., absent and present, fixed) and time (Ti, 11 levels, fixed and crossed with Po), with *n* = 6 per combination of factors, for a total of 132 observation units.

Prior to analysis, the homogeneity of variance was tested using the Cochran test. The Student–Newman–Keuls test (SNK) was used for post hoc comparisons among means [49]. Analysis was done using the GMAV 5 computer program (Statistical software for Windows developed by Institute of Marine Ecology; Marine Ecology Labs, A11, University of Sydney, NSW 2006 AUSTRALIA, 1997).

## 3. Results

### 3.1. Filtration Activity on Amphidinium carterae by Sabella spallanzanii

**Figure 2:**
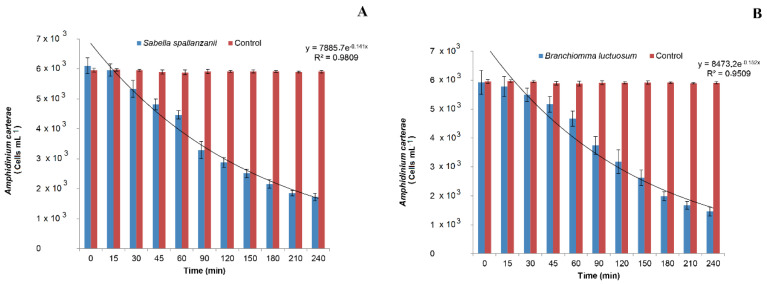
Changes in *Amphidinium carterae* abundances measured in the control and treatment beakers with (**A**) *Sabella spallanzanii* and (**B**) *Branchiomma luctuosum*.

The mean concentrations of *A. carterae* in the control and treatment beakers during the filtration experimental observations employing the polychaete *S.* *spallanzanii* are shown in Figure 2A.

The algal density remained almost unchanged in the control beakers and a mean value of 5.92 ± 0.493 × 10^3^ cells mL^−1^ was measured for the entire duration of the experiment. By contrast, an exponential reduction in phytoplankton concentration was detected in the treatment beakers (y = 7885.7 e^−0.141x^; R^2^ = 0.9809). Analysis of variance revealed a significant Po × Ti interaction (Table 1) and post hoc comparisons among means showed that *A. carterae* concentration in the treatment beakers was always significantly lower than in the control beakers (*p* < 0.01), except for T0 and T15. In particular, the algal density decreased with time in the treatment beakers, starting from T30, when the mean value of 5.33 ± 0.279 × 10^3^ cells mL^−1^ was recorded (*p* < 0.01). The lowest algal concentration of 1.72 ± 0.130 × 10^3^ cells mL^−1^ was measured at time T 240. This last value, however, was not significantly different from the previous one recorded at T 210.

**Figure 3:**
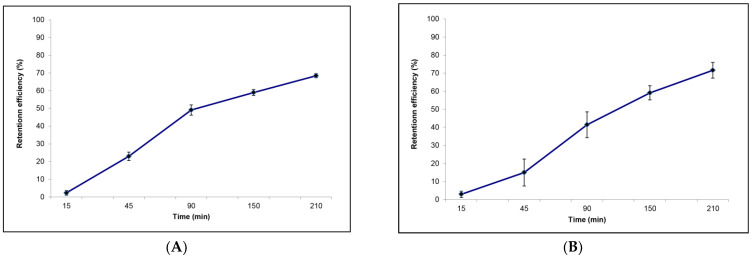
Retention efficiency calculated within each sampling time for (**A**) *Sabella spallanzanii* and (**B**) *Branchiomma luctuosum*.

The retention efficiency of *A. carterae* by *S. spallanzanii* gradually increased with time, with the highest value occurring 210 min after the beginning of the experiment, when the value of R = 68% was recorded (Figure 3A).

**Figure 4:**
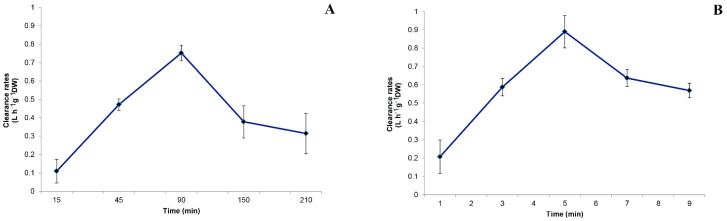
Clearance rates (C) calculated within each sampling time for (**A**) *Sabella spallanzanii* and (**B**) *Branchiomma luctuosum*.

The clearance rate values measured for *S. spallanzanii* within each sampling time are shown in Figure 4A. At T 90, the highest C value was recorded for this species (C = 0.75 L h^−1^ g^−1^ DW), while the lowest value was measured at T 15 (C = 0.11 L h^−1^ g^−1^ DW). The C values significantly differed among sampling times (*p* < 0.001). The C_max_, calculated as the mean value of all the single maximal values recorded for each individual, was 0.936 ± 0.151 L h^−1^ g^−1^ DW.

### 3.2. Filtration activity on Amphidinium carterae by Branchiomma luctuosum

In Figure 2B, the abundance of *A. carterae* recorded at the different experimental times in the control and treatment beakers employing the polychaete *B. luctuosum* is shown.

After the beginning of the experiment, the phytoplankton concentrations in the treatment beakers lowered exponentially as function of time (y = 8473.2 e^−0.152x^; R^2^ = 0.9509). By contrast, the algal concentration in the control beakers remained almost unvaried during the experimental observations, with the mean value of 5.92 ± 0.493 × 10^3^ cells mL^−1^.

The SNK test performed on the Po × Ti interaction after ANOVA (Table 2) revealed that *A. carterae* concentration in the treatment beakers was always significantly lower than in the control ones (*p* < 0.01), except for the initial times of the experimental observations T0 and T15.

In particular, a significant reduction of microalgae in the treatment beakers was observed at T 30 (*p* < 0.01) when the value of 5.49 ± 0.23 × 10^3^ cells mL^−1^ was recorded. At the following sampling times, the algal density further decreased, with time in the treatment beakers reaching the lowest value at T 240 (1.46 ± 0.155 × 10^3^ cells mL^−1^), although no significant difference was detected comparing this value with that recorded at the previous time T 210.

As shown in Figure 3B, the retention efficiency measured for *B. luctuosum* within each sampling time increased with time. At the end of the experimental observations (T 210), the highest value of R = 72% was reached.

The clearance rates calculated for *B. luctuosum* within each sampling time are reported in Figure 4B. The C values ranged from the maximum of 0.86 L h^−1^ g^−1^ DW measured at T 90 min to the minimum of 0.13 L h^−1^ g^−1^ DW at T 15, with significant differences among values over time (*p* < 0.001). The C_max_ calculated as the mean of all the single highest C values measured in each treatment beaker with *B. luctuosum* was 1.15 ± 0.204 L h^−1^ g^−1^ DW.

## 4. Discussion

The problem of harmful algae is crucial due to their effects, including risks for human health, as microalgae are the link in the food chain responsible for the transfer of plankton toxicity to humans [50]. The blooms of harmful microalgae also have a negative influence both on marine resources, directly poisoning wild and farmed fish and invertebrates, and on tourism, because of the effects related to water discoloration and the production of repellent odors and foams. This translates into damage to the marine ecosystem, which inevitably affects the economy of coastal activities and fishing communities directly linked to coastal resources.

The proliferation of potentially harmful microalgae is, therefore, also a problem from an economic point of view. In particular, serious economic losses of several million pounds are caused by HABs in different countries, such as the UK, Australia, and the USA. Thus, the advance in management strategies and mitigation technologies aimed at their removal is of paramount importance in the protection of a significant fraction of the world’s water resources, human health, and economic growth [51,52]. In this framework, phytoplankton filtration by macroinvertebrates could constitute a potential remedy.

This work represents a preliminary attempt to evaluate the ability of the filter feeder polychaetes *Sabella spallanzanii* and *Branchiomma luctuosum* to remove, in relation to their filtration process, a microalgal potentially toxic species, such as *Amphidinium carterae*, representing a danger to human health when present at high concentrations in seawater [36,37,38].

From our results, some interesting issues can be inferred.

- The potentially harmful microalgal species *A. carterae*, here used, when present in the surrounding environment, can be an integral part of the natural diet of the two investigated polychaetes. The C_max_ and highest retention efficiencies obtained are 0.936 ± 0.151 L h^−1^ g^−1^ DW and 68% for *S. spallanzanii*, and 1.15 ± 0.204 L h^−1^ g^−1^ DW and 72% for *B. luctuosum*, respectively.

Clearance rates of other polychaete species were always estimated by using phytoplankton in laboratory and field experiments [22,23,24,29,30,33,34,53,54,55,56]. In particular, the filtering activity of *S spallanzanii* has been investigated by Clapin [29] and Lemmens et al. [56], who reported that this species has a filtering capacity similar to that of the macro-filter-feeder community inhabiting the *Posidonia sinuosa* meadows of Southern Flats in Cockburn Sound (Australia). They estimated a mean filtering capacity of about 12 m^3^ d^−1^ m^−2^ habitat and a feeding efficiency of 13 L mg^−1^ O_2_ consumed.

Clearance rates on phytoplankton have also been recorded by employing other filter feeders. For example, due to their importance from an economic point of view, a group of filter feeders that has been widely studied in this regard is represented by mussels. From a paper by Cugier et al. [57] on the filtration capacity of phytoplankton of some bivalves, it appears that the clearance rates of these organisms vary from a minimum of 0.1 L h^−1^ g^−1^ DW to a maximum of 4 L h^−1^ g^−1^ DW. Thus, because of these considerations, it is clear that filter feeders, including polychaetes, play a crucial role and have a significant impact on the concentration of phytoplankton in their surrounding environment.

It is also important to underline that filter feeder polychaetes play a very crucial role in regulating the abundance of some components of the ecosystem, which not only include microalgae, as demonstrated by the present study, but also bacteria. In particular, some studies undertaken in order to investigate the effect of the filtration activity of *S. spallanzanii* and *B. luctuosum* on bacterioplankton density showed that the clearance rates and retention efficiencies for these two sabellid species were 12.4 L h^−1^ g^−1^ DW and 70% for the former, and 43.2 L h^−1^ g^−1^ DW and 98% for the latter [14,15,16]. The different values of clearance rates obtained using bacteria or phytoplankton as trophic sources is not surprising since several studies have shown that clearance rate values vary in relation to the size of the particles used. The recorded values of clearance rate obtained in the present study are particularly remarkable as a phytoplanktonic component with dimensions around 12–18 µm in length and 8–10 µm in width was supplied to the polychaetes. *Amphidinium carterae*, indeed, was considerably larger than bacteria that also constitute a significant constituent of the polychaetes’ diet. Furthermore, several works have shown that the higher the concentration of suspended particles (algal cells, etc.), the lower the filtering activity and the food pressure on the plankton [11]. This means that, at relatively low particle concentrations, the relationship between the filtration rate and the quantity of particles is linear, or almost linear. Above a certain concentration threshold, however, this relationship becomes non-linear, [25]. Based on these considerations, presumably employing different concentration of *A. carterae* could give rise to a variability in the filtering activity on phytoplankton, leading to a relative decrease in the abundance of harmful algae in aquatic ecosystems.

- The here-obtained results on the filtration capability of the polychaetes *S. spallanzanii* and *B. luctuosum* on the harmful dinoflagellate *A. carterae* suggest that these invertebrates represent a new tool to reduce the impact of harmful algae on marine life and could constitute a potential advantage in comparison to the up-to-now used bioremediation methodologies in the bioremediation process. Current strategies to reduce the potential economic and human health effects from HABs to fisheries and aquaculture include chemical (e.g., flocculation [43]), physical, and biological measures [4]. Among these strategies, flocculation has been classified as the most cost-effective and convenient way to rapidly remove algae [58,59,60]. Flocculation by the sole use of natural clay, however, requires a high dosage in order to attain a fairly high (> 90%) removal efficiency [61,62]. New methods were tested to further increase the removal efficiency of natural clay minerals for the mitigation, for example, of *A. carterae* [41,43], but in general, the use of clay remains often too rudimental, confined, or problematic for large-scale implementation and could potentially have detrimental effects on filter-feeding invertebrates [63,64]. For these reasons, in many countries, severe environmental controls preclude the use of these techniques [3]. As regards physical methods, often used to minimize HABs’ impacts on aquaculture plants, they consist, for example, of enhanced flushing and aeration of cages and/or feeding cessation that would help to manage the problem, but only in a small volume of water [10]. Finally, biological methods are environmentally friendly and can be specific for algal cells, without impact on non-HAB species. These include the use of bacteria, fungi, phages, macroalgae or seagrass, protozoa, zooplankton, and filter-feeding shellfish [4].

The employment of these two species in the control of harmful algae could also be recommended in the case of aquaculture production, where HABs are one of the most critical threats. Evidence on the occurrence and impacts of HABs on marine fisheries and mariculture is being gathered by ongoing regional programs (e.g., [65]), national programs (e.g., UK FSA, https://www.food.gov.uk/business-guidance/biotoxin-and-phytoplankton-monitoring, accessed on 9 December 2021), and global programs (Global HAB Status Report) [66,67]. In the Gulf of Taranto, we realized an integrated multitrophic aquaculture (IMTA) system involving these polychaetes and macroalgae as bioremediators, reared/cultivated in association with fish cages, obtaining a high production of bioremediator biomass. At the same time, a restoration of the aquaculture-rearing environment was achieved in terms of microbial contamination (i.e., total coliforms and *Escherichia coli*) and nutrient concentrations (i.e., phosphorous and nitrogen salts were also realized) [68], as well as an amelioration of the benthic communities under the cages. It is noteworthy to underline that *B. luctuosum* is not originally in the Mediterranean Sea and can be considered an invasive species. However, in the long term, this species has reached a “naturalized” condition, so becoming a part of the fouling community everywhere along the Mediterranean coast without any negative impact [69]. The here-obtained results represent an added value indicating the potentiality of the reared polychaetes to remediate the surrounding farming environment also in terms of harmful algae, thus contributing to the realization of an eco-friendly approach. This is useful to guarantee food safety and environmental quality, taking into account that managing global food security is one of the greatest challenges of the twenty-first century. The realization of an IMTA system including polychaetes is also noteworthy considering that, despite the many reported cases of laboratory success in controlling HABs, only a few field applications are described [70]. In fact, there are great difficulties in cultivating and transporting the control agents when these technologies are applied in the water, and they cannot be applied in the case of a rapid response to HAB events. Therefore, as already suggested by Yu et al. [4], the main challenge of the biological control for HABs mitigation is to transfer the laboratory experiments into the environment that seems achievable in the case of the two considered polychaete species.

## 5. Conclusions

In conclusion, although our results are preliminary, they indicate a conspicuous filtration capability of the toxic microalga *A*. *carterae* by the two considered filter feeder polychaetes *S. spallanzani* and *B. luctuosum*. However, further studies will be carried out to confirm these results in the field. Furthermore, in the future, other experiments will be conducted using other typically planktonic algal species of different sizes to evaluate the effectiveness of the proposed technology in different conditions. 

Since harmful algae have become an important global issue, the idea of employing the two considered polychaetes as bioremediatiors for purification of the water column is a sustainable, eco-friendly novelty also considering that, over the past several decades, researchers have made great efforts to develop an integrated management approach for HABs control.

The employment of *S. spallanzanii* and *B. luctuosum* in the control of HABs could be recommended not only also in the case of the bioremediation of marine ecosystems, but also in aquaculture facilities where HABs are one of the most critical threats.

## Figures and Tables

**Table 1:** Summaries of ANOVA testing for differences in average *Amphidinium carterae* abundances measured at the different sampling times in the control and treatment beakers with *Sabella spallanzanii*.

Source of Variation	DF	MS	F	*p*
Po	1	157,564,720.3712	6543.83	***
Ti	10	8,376,261.4636	347.88	***
Po × Ti	10	8,036,449.4879	333.76	***
Residual	110	24,078.35		
TOT	131			
Cochran Test Transformation	C = 0.1854 (*p* < 0.05) None			
SNK Test	AT < AC			
Po(Ti)	(AT T0 = AC T0)			
	(AT T1 = AC T1)			
	AT T0 > AT T1 > AT T2 > AT T3 > AT T4 > AT T5 > AT T6 > AT T7 > AT T8 > AT T9 > AT T10			
Ti(Po)	(AT T10 = AT T11)			
	AC T0 = AC T1= AC T2 = AC T3 = AC T4 = AC T5 = AC T6 = AC T7 = AC T8 = AC T9 = AC T10 = AC11			

Reported are: Po: polychaetes; Ti: time; AT: algal concentration in the treatment beakers; AC: algal concentration in the control beakers; AT T0: algal concentration in the treatment beakers at T0; AT T1: algal concentration in the treatment beakers at T1; AT T*n*: algal concentration in the treatment beakers at T*n* (with *n* = 1,2,3……11); AC T0: algal concentration in the control beakers at T0; AC T1: algal concentration in the control beakers at T1; AC T*n*: algal concentration in the control beakers at T*n* (with *n* = 1,2,3……11); ***: *p* < 0.001.

**Table 2:** Summaries of ANOVA testing for differences in average *Amphidinium carterae* abundances measured at the different sampling times in the control and treatment beakers with *Branchiomma luctuosum*.

Source of Variation	DF	MS	F	*p*
Po	1	149,460,080.9	2990.75	***
Ti	10	8,872,472.823	177.54	***
Po × Ti	10	8,561,803.017	171.32	***
Residual	110	49,974.1591		
TOT	131			
Cochran Test Transformation	C = 0.1902 (*p* < 0.05) None			
SNK Test	AT < AC			
Po(Ti)	(AT T0 = AC T0)			
	(AT T1 = AC T1)			
	AT T0 > AT T1 > AT T2 > AT T3 > AT T4 > AT T5 > AT T6 > AT T7 > AT T8 > AT T9 > AT T10			
Ti(Po)	(AT T10 = AT T11)			
	AC T0 = AC T1= AC T2 = AC T3 = AC T4 = AC T5 = AC T6 = AC T7 = AC T8 = AC T9 = AC T10 = AC11			

Reported are: Po: polychaetes; Ti: time; AT: algal concentration in the treatment beakers; AC: algal concentration in the control beakers; AT T0: algal concentration in the treatment beakers at T0; AT T1: algal concentration in the treatment beakers at T1; AT T*n*: algal concentration in the treatment beakers at T*n* (with *n* = 1,2,3……11); AC T0: algal concentration in the control beakers at T0; AC T1: algal concentration in the control beakers at T1; AC T*n*: algal concentration in the control beakers at T*n* (with *n* = 1,2,3……11); ***: *p* < 0.001.

## Data Availability

Not applicable.
